# Survival rate of the Hall technique compared with resin composite restoration in multi-surface cavities in primary teeth: a 1-year randomized clinical trial

**DOI:** 10.1590/1678-7757-2023-0048

**Published:** 2023-10-09

**Authors:** Aline Maquiné PASCARELI-CARLOS, Tamara Kerber TEDESCO, Ana Flávia Bissoto CALVO, Isabela FLORIANO, Thais GIMENEZ, Monicque da Silva GONÇALVES, Daniela CALUMBY, José Carlos Pettorossi IMPARATO

**Affiliations:** 1 Universidade Cruzeiro do Sul Programa de Pós-graduação em Odontologia São Paulo Brasil Universidade Cruzeiro do Sul, Programa de Pós-graduação em Odontologia, São Paulo, Brasil.; 2 Instituto São Leopoldo Mandic e Centro de Pesquisa Programa de Pós-graduação em Odontologia Campinas Brasil Instituto São Leopoldo Mandic e Centro de Pesquisa, Programa de Pós-graduação em Odontologia, Campinas, Brasil.; 3 Universidade Centro Santo Agostinho Teresina Brasil Universidade Centro Santo Agostinho, Teresina, Brasil.; 4 Universidade Metropolitana de Santos Santos Brasil Universidade Metropolitana de Santos, Santos, Brasil.

**Keywords:** Tooth, deciduous, Dental caries, Composite resins, Hall technique

## Abstract

**Background:**

Hall technique (HT) has been indicated for teeth with dentinal caries lesion; however, extensive cavities, with more than two surfaces still seem challenging for restorative treatment in pediatric dentistry, resulting in a higher failure rate and an increased need for retreatment.

**Objectives:**

To compare the survival rate of the Hall technique preformed metal crown (HT) with resin composite restoration (RC) for multi-surface cavitated caries lesions in primary molars.

**Methodology:**

In this multicenter two-arm randomized clinical trial, children between 4 and 9 years of age with at least one primary molar with cavitated caries lesion involving more than two surfaces, including one buccal or palatal/lingual surface, were selected from 17 Brazilian cities. A total of 364 teeth were allocated into two groups: (1) teeth treated with selective caries removal and RC and (2) treated with the HT. The survival rate was assessed at 6 and 12 months after the interventions. Survival analysis was performed with the Kaplan‒Meier method. Cox regression was used to determine the influence of explanatory variables on the survival rate (α=5%).

**Results:**

After 12 months, 292 teeth were re-evaluated. A total of 358 teeth were re-evaluated at least once during the study and included in the survival analysis. The HT (87.8%) resulted in a higher survival rate than RC restoration (75.7%) (p=0.004).

**Conclusion:**

HT has a higher survival rate than RC as a treatment for multi-surface cavitated caries lesions in primary teeth. ClinicalTrials.gov: NCT02782390

## Introduction

Untreated caries lesions remain one of the main prevalent disease conditions worldwide.^1^ Despite the strategies to try to control its establishment and progression, dental caries is still a public health problem that could evolve into cavitated caries lesions.

Cavitated caries lesions that involve more than two surfaces, including proximal, occlusal, and smooth surfaces presents a challenge for rehabilitation, since extensive cavities could result in a higher failure rate and an increased need for retreatment,^2^ which directly impacts the cost-effectiveness of the intervention.^3^ Furthermore, diagnosing the pulp condition in this type of cavity can also be challenging, since the radiographic image may not accurately assess the dentin between the cavity floor and the pulp chamber when the buccal or palatal/lingual surface is involved.^4^

Cost-effective, patient-friendly, and accessible approaches have been developed and target the biological control of the dental caries without major invasive interventions as practiced heretofore.^5-8^The Hall technique is one of these possible approaches to treat cavitated or non-cavitated dentine caries lesions in primary dentition molars.^8-11^ The scientific evidence confirms its positive outcomes on cavitated caries lesions involving occlusal and occluso-proximal surfaces.^5,8,9^ Systematic reviews consider this treatment the best option for occluso-proximal cavities^10^ and deep caries lesions in primary teeth.^11^

Conversely, to the best of our knowledge, no randomized clinical trials have focused on the survival rate of the Hall technique in cavitated caries lesions that involve more than two surfaces, including proximal, occlusal, and smooth surfaces, when compared with other materials such as the conventional restorative technique. Thus, this study aimed to compare survival rate of the Hall technique (HT) with resin composite restoration (RC) in multi-surface cavitated caries lesions in primary molars.

## Methodology

### Trial design and ethical approval

This was a multicenter randomized controlled clinical trial, with two-arm parallel groups (1:1 allocation rate) with one year of follow-up, registered in the clinical trials database (ClinicalTrials.gov: NCT02782390). The secondary outcomes foreseen in the protocol will be further analysed and published. The manuscript was reported following the Consolidated Standards of Reporting Trials (CONSORT) guideline.^12^ The study protocol was approved by the Research Ethics Committee of Faculdade São Leopoldo Mandic. Legal guardians of participants signed written informed consent forms, and the children indicated their participation by verbal agreement.

### Participants

Children aged between 4 and 9 years old from public and private dental clinics in 17 cities in the five regions of Brazil – Amargosa-BA, Aracajú–SE, Ariquemes-RO, Brasília-DF, Botucatu-SP, Cacoal-RO, Chopinzinho-PR, Curitiba-PR, Goiânia-GO, Gurupi-TO, Itagibá-BA, Livramento de Nossa Senhora-BA, Manaus-AM, Rio Branco-AC, São Félix do Xingu-PA, Uberlândia-MG, Umuarama-PR – were selected. Patients were recruited as they appeared for screening sessions. The selection of cities was by convenience, due to the patient selection sites being close to the researchers’ location. On average, each researcher included approximately 20 participants, since not all researchers could include all the requested children in the study.

Children with at least one primary molar with multi-surface cavities lesions, who were cooperative during the screening, and 6 and/or 12-month follow-up, were included. Multi-surface cavities were defined as those involving more than two surfaces, including proximal, occlusal, and smooth surfaces (buccal or palatal/lingual surface), without pulp involvement. Patients with special needs or systemic diseases with oral impairment were excluded. In addition, teeth with spontaneous pain; pulp exposure, i.e., absence of dentin between caries lesion and pulp chamber; or other signs related to pulp necrosis, such as periapical or interradicular radiolucent lesions, or presenting restorations, sealants, or enamel developmental defects were excluded.

### Interventions

After a clinical and radiographic examination, the teeth of the children who met the eligibility criteria, i.e., those with multi-surface cavities lesions, were randomly allocated into two parallel groups: teeth with resin composite restoration or teeth treated with the Hall technique. The trial setting was private and public dental offices.

A total of 20 operators, specialists in pediatric dentistry, one from each city (except for Manaus, Brasília, and Goiânia, which had two operators each), participated in all stages of the children’s evaluation and intervention. They underwent theoretical-practical training for both interventions in Campinas/SP. The training included a theoretical-laboratory class that covered the ICDAS (International Caries Detection and Assessment System) classification, as well as the step-by-step process of resin composite restoration (RC) and crown installation. It comprised three hours of theoretical and laboratory classes and four hours of clinical treatment of patients with characteristics similar to those of the eligible children. Each operator treated one RC patient and one HT patient, resulting in a total of thirty-eight treated patients. Operators were evaluated based on the accuracy of their diagnoses and their adherence to the treatment protocol specified for each treatment. Before the interventions were performed, participant’s socioeconomic data, the characteristics of the teeth included (jaw and tooth – first or second molar), and the cavity volume of the teeth were recorded individually. The socioeconomic data were collected by the dentist with a questionnaire, which included only variables such as gender and age. The cavity volume was considered the multiplication of the mesiodistal, occlusal-cervical, and buccolingual/palatal measurements.

Participants with teeth allocated to the restoration with RC group initially received dental prophylaxis, local anesthesia, and rubber-dam isolation. Selective caries removal to soft dentin was then performed. Etching with 37% phosphoric acid (Condac 37, FGM; Joinville, BRA) was performed for 15 s for both the enamel and dentin, followed by the application of the two-step etch-and-rinse adhesive system (Adper Single Bond^®^, 3 M/ESPE; Minnesota, USA), according to the manufacturer’s instructions. A 5 mm stainless steel matrix band system (Microdont, São Paulo, Brasil) was used and the cavity was then restored with resin composite by the incremental technique (Z250^®^, A1, 3 M/ESPE; Minnesota, USA), and each 2-mm increment was photoactivated for 20 s.

Participants with teeth allocated to the Hall technique (HT) group were treated according to a previously published protocol.^13^ Initially, the orthodontics separators were used for 48 hours to make it possible to fit the crown. The preformed stainless-steel crown (3M/ESPE; Minnesota, USA) was chosen according to the tooth size and then adjusted using a tungsten drill and band-forming pliers. After dental prophylaxis, the airway was protected with gauze, and the crown was loaded with glass ionomer cement (Fuji Plus C^®^, GC Corporation; Tokyo, JPN). The crown was then seated in the tooth, and the participant was instructed to bite a cotton roll on the occlusal surface. Then, the excess cement was cleared.

X-rays of all teeth were performed immediately after the intervention and 6 and 12 months similarly. All participants received oral hygiene and diet instructions (mainly based on a reduction in the frequency of sugar intake). Other teeth with intervention needs were treated by the operators but were not included in the study.

### Follow-up and Outcome

The primary outcome of this trial was the survival of the interventions. Participants were scheduled for clinical examination 6 and 12 months after the interventions. They were reminded of their follow-up visits by phone calls or messages 30, 15, and 7 days prior to the scheduled return date. Additionally, during the month of the return, efforts were made to reschedule any missed appointments. Participants who could not attend were considered dropouts for follow-up. The examiners and operators of the interventions were the same. The training consisted of three hours of theoretical lectures with photograph evaluations.

The criterion proposed by Innes, Evans, Stirrups^9^ (2011) was used to evaluate the primary outcome. Restorations with resin composite and the Hall technique were considered a “success” when they appeared satisfactory (entire tooth surface adjacent to restoration, stained margins consistent with non-carious lesions), did not require intervention, did not show clinical signs or symptoms of pulpal pathology, and did show signs of physiologic tooth exfoliation.

### Sample size

For sample size estimation, due to the lack of previous studies evaluating the survival rate of a resin composite in cavities involving more than two surfaces, we considered data from restorations on two surfaces. Thus, a 73% expected survival rate was considered for resin composite restorations after 12 months of follow-up.^14^ A clinically significant 15% increase in the survival rate in the Hall technique group, a significance level of 0.05, and a power of 0.80 were adopted. Considering a two-tailed test, adding a 10% attrition rate and a further 40% due to the study design (multicenter), a final number of approximately 364 total teeth was reached (G*Power 3.1.3; Düsseldorf, DE).^15^

### Randomization and allocation concealment

The teeth were allocated to two parallel arms: the RC and HT groups. A sequence of random numbers was generated by an external researcher, considering the city as strata in blocks of 4 or 6, obtained from www.sealedenvelope.com. The group allocation was concealed in sealed, numbered opaque envelopes, opened by the operators only at the time of intervention. The experimental unit in the study was the tooth, and only one tooth per patient was included. In case where participants had more than one eligible tooth, each tooth meeting the inclusion criteria was assigned a unique number. These numbers were written on paper, folded, and placed in an opaque envelope. An independent dentist, not involved in the research, was responsible for selecting one envelope containing the tooth number to be included in the study.

### Blinding

Blinding of the patients, operators, and examiners was not possible due to the difference between the interventions. However, the statistical advisor was blinded regarding the groups.

### Deviations from the protocol

Initially, the evaluation was planned with two different criterions for resin composite restoration and Hall technique; however, we deviated from the protocol and used the exact same “success” criterion for both interventions. This was necessary to facilitate comparability.

### Statistical methods

Kaplan‒Meier curves were used to estimate the survival of the interventions. The log-rank test evaluated the differences between the survival curves. Participants assessed at least once during the study were included in the analysis. The multivariate Cox regression model was used to determine the possible influence of explanatory variables on the failure of the interventions. Initially, an unadjusted analysis was performed for each explanatory variable, and those with p≤0.20 were tested into the adjusted analysis. The final model included variables with a p≤0.05. Hazard ratios (HRs) and 95% CIs were calculated. Proportional hazard assumption in the Cox model was tested using Schoenfeld residuals.

Intention-to-treat analysis (ITT) using multiple imputation was conducted considering the proportion of intervention success at 12-month follow-up. Odds ratios (OR) and 95% confidence intervals (CI) were calculated.

Statistical analysis was performed using the RStudio, version 1.1.45 statistical software, version 4.0.2 (R Core Team, 2012, Vienna, AUT). The significance level was set as 5% for all analyses.

## Results

We randomly allocated 364 teeth for treatment with RC restoration (n=182) or HT (n=182). Children were enrolled by sequential recruitment in the trial between February 2016 and December 2016, and the final follow-up assessment took place in December 2017. Table 1 shows the number of patients included per city.

**Table 1:** Number of patients included per city and dental service

CITY	SCREENING PATIENTS	PATIENTS INCLUDED	PUBLIC OR PRIVATE DENTAL SERVICE
Amargosa-BA	100	20	PUBLIC
Aracajú–SE	75	20	PUBLIC
Ariquemes-RO	154	20	PRIVATE
Brasília-DF	120	40	PUBLIC
Botucatu-SP	125	20	PUBLIC
Cacoal-RO	51	20	PRIVATE
Chopinzinho-PR	370	20	PUBLIC
Curitiba-PR	50	1	PRIVATE
Goiânia-GO	125	39	PUBLIC
Gurupi-TO	218	10	PRIVATE
Itagibá-BA	114	20	PRIVATE
Livramento de Nossa Senhora-BA	100	20	PUBLIC
Manaus-AM	320	40	PUBLIC
Rio Branco-AC	56	20	PRIVATE
São Félix do Xingu-PA	159	20	PRIVATE
Uberlândia-MG	240	20	PUBLIC
Umuarama-PR	54	14	PRIVATE
TOTAL	2.744	364	

**Figure 1:**
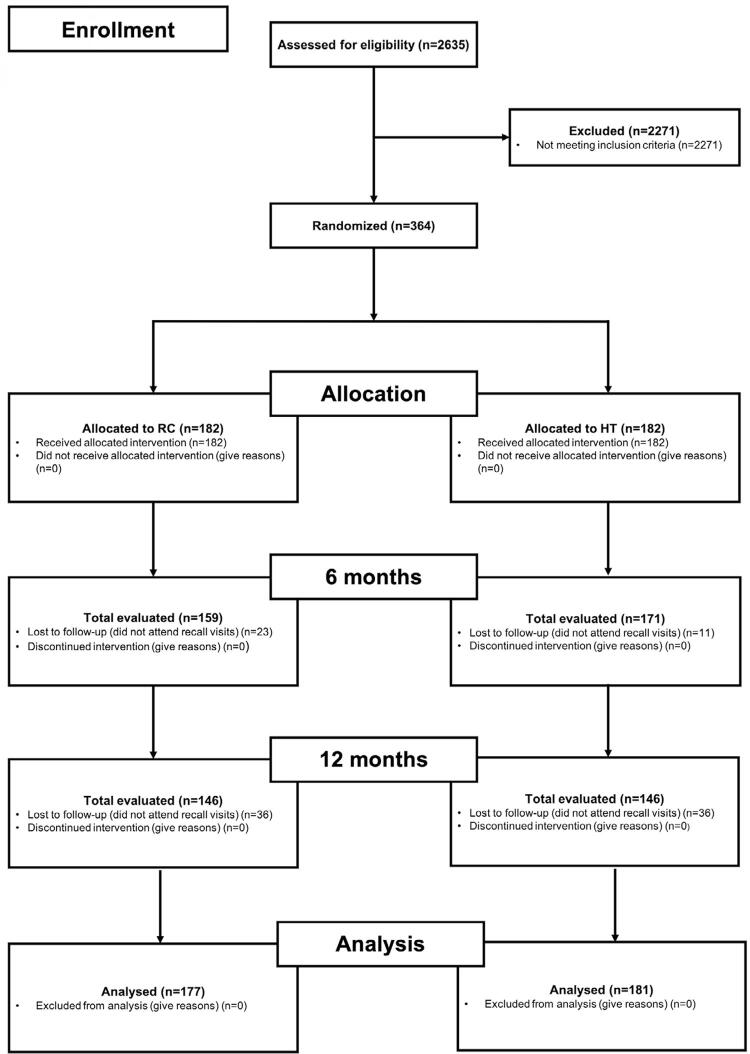
Flow chart of the participants across the trial phases

After 6 months, 330 teeth (90%) were re-evaluated (RC – 159; HT – 171). After 1 year, 292 teeth (80%) were re-evaluated (RC – 146; HT – 146). In total, 358 teeth were evaluated at least once during the study. We found no difference between the groups in the number of participants analyzed at the beginning and the end of the trial (p=0.880). Figure 1 shows the flowchart of participants across the trial phases.

Table 2 shows the baseline characteristics of the participants according to the groups. In both groups, most participants were male (HT=60%; RC=51.1%), with a mean age of 6.85 (±1.40) years and a high caries experience (dmft+DMFT=6.17±3.46).

**Table 2:** Baseline characteristics of the participants at baseline according to the groups

	Group		
Characteristics	HT	RC	Total
Sex n(%)			
Female	73 (40%)	89 (48.9%)	162 (44.5%)
Male	109 (60%)	93 (51.1%)	202 (55.5%)
Age - Mean (SD)	6.9 (1.6)	6.8 (1.2)	6.85 (±1.40)
dmft + DMFT - Mean (SD)	6.6 (3.4)	5.7 (3.53)	6.17 (±3.46)

**Figure 2:**
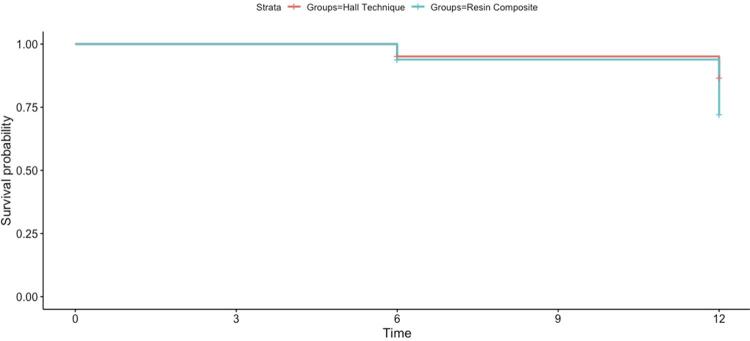
Kaplan‒Meier survival curve of the HT and RC groups throughout the 12-month follow-up period

Figure 2 shows the Kaplan‒Meier curve for both groups. The HT resulted in a higher survival rate (87.8%) than RC restoration (75.7%) after one year of follow-up (p=0.0028).

We could observe nine failures for the RC group and eight for the HT group after six months. After one year, we observed 29 failures for RC and 13 for HT.

Table 3 shows the results of the Cox regression analysis. The covariates tests and the global test were not statistically significant using conventional p-value thresholds. Therefore, the proportional hazards assumption was not violated. Only the intervention group and age influenced the failure of the interventions. Teeth that received RC restoration had a 2.19 times higher chance of failure than those treated with HT. Moreover, children with ≥7-year-old had a 38% lower chance of failure of intervention than the younger ones.

**Table 3:** Cox regression analysis (hazard ratio; 95% confidence interval) for the failure of restorations according to explanatory variables

Variables		Survival N (%)	Unadjusted HR (95% CI)	p-value	Adjusted HR (95% CI)	p-value
Group	HT	159 (87.8%)	Ref.	0.003	Ref.	0.003
	RC	134 (75.7%)	2.171 (1.298-3.629)		2.198 (1.31-3.67)	
Sex	Female	130 (80.7%)	Ref.	0.590		
	Male	163 (82.7%)	0.874 (0.537-1.423)			
Age	≤ 6-year-old	131 (78.4%)	Ref.	0.059	Ref.	0.049
	≥ 7-year-old	162 (84.8%)	0.624 (0.382-1.019)		0.612 (0.38-0.99)	
Jaw	Upper	96 (79.3%)	Ref.	0.382		
	Lower	197 (83.1%)	0.800 (0.485-1.319)			
Tooth	First molar	153 (80.1%)	Ref.	0.248		
	Second molar	140 (83.8%)	0.749 (0.457-1.227)			
Cavity-volume Mean (SD)	42.9 (42.8)		0.996 (0.989-1.003)	0.257		

Table 4 shows the results of an ITT analysis of the primary outcome. The RC group showed a higher chance of failure at the 12-month follow-up.

**Table 4:** Intention-to-treat analyses of the primary outcome

	Group		
**Outcome**	**HT**	**RC**	**p-value**
N success/N total	159/182	134/182	
% Success	87.4%	73.6%	0.001
OR (95% CI)	2.476 (1.432 - 4.282)		

### Harms or unintended effects

No harm or unintended effects were observed or reported in either group.

## Discussion

The HT is considered the best treatment option for cavitated caries lesions in primary molars.^2,8,16^ Still, the question of an effective treatment for multi-surface cavities with more than two surfaces remains unanswered. Thus, our study evaluated the survival rate of HT compared with RC restoration in this scenario. After a 12-month follow-up, the HT group showed a higher survival rate. Our positive result can be explained by the dynamics involved in the progression of caries lesions. The role of the pre-formed crowns (PMC) in isolating the microorganisms from biofilm in a caries lesion and preventing their interaction with sucrose from a patient’s dietary intake has been highlighted.^2,6^ Thus, the microorganisms can be trapped inside the cavity without caries lesion progression.^6^ Furthermore, for direct restorative treatment, a higher number of surfaces involved could increase the failure rate due to the longer treatment time and number of steps involved, such as dentin conditioning, which is extremely sensitive to error; consequently, retreatment may be needed.^2^

In an earlier study, Araujo, et al.^8^ (2020) reported a HT survival rate almost three times higher (93.4%) than that of the atraumatic restorative treatment (ART) (32.7%) in treating occluso-proximal cavities over 36 months, corroborating other survival data in which the HT showed better results than the different treatment options evaluated.^5,6,16-19^ Although our results for the HT were also significantly superior to those for RC restoration (88% and 76%, respectively), the lower survival rate observed compared with previous studies may be explained by the type of cavities included. More extensive cavities can lead to initial pulp diagnosis errors. Especially in primary teeth, diagnosing pulp health is difficult due to the invisible characteristics of pulp degeneration and necrosis, as well as the limitation of reports of spontaneous pain from children.^20^ Thus, a possible reason for the failure of HT could be related to the erroneous indication of the technique by the operators, with the children without spontaneous pain at the time of diagnosis and the pulpal communication not being radiographically visualized. The difficulty in diagnosis, particularly related to the reporting of pain, could also explain the age-related differences, since younger children may have more difficulty reporting pain data.

Furthermore, we are aware of the limitations of both techniques. In addition to those regarding composite resin, in cavities involving more than two surfaces, polymerization contraction stress, lack of marginal adaptation, and the presence of gaps can lead to failures.^21^ The HT, due to its installation technique, appears to eliminate such problems. However, if the operator fails to properly install it, resulting in a lack of adaptation and complete sealing, a risk of carious progression due to continuous bacterial communications with the substrate is possible.^16^

In addition, in reviewing previous randomly controlled trials (RCTs) published about the HT survival rate, different follow-ups have been considered, ranging from 6 to 72 months.^6,8,9,22,23^ The follow-up time of treated patients is important to confirm the technique’s success. Whereas an ideal time frame for follow-up would be until tooth exfoliation, a 12-month follow-up provides an important view of the behavior of the HT in treating multi-surface cavities with more than two surfaces compared with RC restoration, considering the biological cycle of primary teeth. The ideal follow-up would likely increase the research cost without producing much additional information concerning the technique’s effectiveness.^24^ However, we do not expect to find different results regarding the higher survival rate of HT compared with RC. On the contrary, with a longer follow-up period, we may observe an even greater difference in survival rates between the two interventions.

The sample size calculation was based on a previous study that evaluated the survival rate of resin composite in only occluso-proximal cavities,^14^ which is different from our study that aimed to evaluate the performance of strategies in multi-surface cavities, including smooth surfaces. However, no previous studies at the start of this RCT evaluated resin composite in cavities with more than two surfaces, including a smooth surface. Therefore, the sample size may have been underestimated. Nevertheless, we could identify a significant difference between the groups, indicating that our sample size was sufficient to compare both interventions.

Another relevant point is that the examiners of the outcomes were also the operators providing the interventions, which could introduce a detection bias. However, even if the examiner was a different researcher who did not perform the intervention, the difference between the interventions does not change the possibility of blinding for the outcome assessment. Moreover, we did not perform the intra-examiner and inter-examiner reliability of the assessments, which may affect the validity and reproducibility of the results. However, as previous mentioned, the examiners of outcomes received the training for the criterion used to evaluate the success of restorations.

The multicenter design of this study also resulted in diverse operators performing the interventions, resulting in another possible limitation due to the operational difference of each dentist.^25^ However, all the operators, who were specialists in pediatric dentistry, received theoretical-practical training for both interventions. Also note that selecting participants from different cities covering five regions of Brazil with diverse customs certainly made carrying out this research challenging. However, including participants selected from both public and private dental service, from different cities means that the results of this study could reflect the behavior of HT in more than two surface cavities in primary molars of children from diverse cultural and sociodemographic backgrounds.

In Brazil, we have a monetary difference between regions. Previous studies have reported low income as an additional factor in caries risk due to difficulty accessing the dentist and oral hygiene products.^26^ The South and Southeast regions have the highest percentage of children aged 12 years free from caries. Whilst the North and Northeast regions, which have some of the poorest states in the country, have the highest rates of decayed, not physiologically missing, and obturated teeth since the first general oral epidemiological data in 1986, continuing in 2003 and 2010.^27-29^ Such evident differences between regions included in this research may show a different behavior of treatment strategies.

Thus, due to HT high survival rate, this technique can be indicated as a treatment type for multi-surface cavities with more than two surfaces involved in primary molars. Although our study did not report cost analysis, which will be performed as a secondary outcome, note that the HT results in lower financial expenditure.^30^ Studies that evaluated the cost-effectiveness of the HT confirm this, since fewer dental office visits are needed to provide repairs/replacements or further treatments.^30,31^

In Brazil, approximately 75% of the population uses the Unified Health System, which encompasses children’s dental care.^32^ This is one more reason for having good material available for managing caries, but for this to happen, scientific evidence needs to reach our government officials. They also need to know the importance of funding for training in these cases, so that pediatric dentists can improve their care. As mentioned in the previous literature,^33^ although the number of dentists working in the SUS has increased, patient/professional demand is still disproportionate, thus in addition to improving the quality of care provided by the professional, implementing HT would benefit children and could reduce the amount spent on treatments for this health condition. However, studies exploring the facilitators and barriers to implementing HT for public health services must confirm these benefits.

Moreover, further randomized clinical trials focusing on approaches to treating cavities involving more than two surface are suggested, since this still seems to be an obstacle to restorative treatment in pediatric dentistry. However, the higher survival rate for HT compared with CR at 1-year follow-up is positive for this purpose.

## Conclusion

Hall technique has a higher survival rate than RC as a treatment for multi-surface cavities with more than two surfaces, including one buccal or palatal/lingual surface for caries lesions in primary teeth.
